# Prenatal Sonographic Features of Cri-du-Chat Syndrome: A Case Report and Analytical Literature Review

**DOI:** 10.3390/diagnostics12020421

**Published:** 2022-02-06

**Authors:** Kuntharee Traisrisilp, Yuri Yanase, Srimeunwai Ake-sittipaisarn, Theera Tongsong

**Affiliations:** 1 Department of Obstetrics and Gynecology, Faculty of Medicine, Chiang Mai University, Chiang Mai 50200, Thailand; kuntharee.t@cmu.ac.th (K.T.); conan227kku@gmail.com (S.A.-s.); 2 Department of Obstetrics and Gynecology, Nakornping Hospital, Chiang Mai 50180, Thailand; yuletides@hotmail.com

**Keywords:** Cri-du-Chat syndrome, prenatal diagnosis, ultrasound

## Abstract

Cri-du-Chat syndrome (CdCS) is a rare but serious genetic disorder. Most cases occur de novo, without specific risk factors as an indication of invasive prenatal diagnosis. Moreover, no specific ultrasound findings have been reported to facilitate early detection. This study presents a case of CdCS with fetal ultrasound findings of cerebellar hypoplasia and peri-membranous ventricular septal defect (VSD), which are consistent with previous reports, as well as coarctation of the aorta and hypercoiling cord, which have never been described in CdCS before. Additionally, we performed an analytical literature review to identify the sonographic pattern facilitating prenatal diagnosis. Based on the review of 47 reported cases, most CdCS fetuses (87.2%) had ultrasound characteristics: cerebellar hypoplasia (29.8%), followed by cardiac abnormalities (19.1%), hydrops fetalis/fluid collection (17.0%), ventriculomegaly (14.9%), choroid plexus cyst (12.8%) and nasal bone hypoplasia (12.8%). Increased nuchal translucency/nuchal fold thickness was also common. This is the first study providing a fetal sonographic pattern of CdCS that may facilitate early diagnosis.

## 1. Introduction

Cri-du-Chat syndrome (CdCS), or cat-like-cry syndrome, was first described by Lejeune in 1963 [1]. It is a rare genetic disorder with an incidence of 1:15,000–1:50,000 live births [2], but it is one of the most common chromosomal deletion syndromes. It results from the short arm of chromosome 5 deletion (5p-). It is characterized by microcephaly, micrognathia, and a high-pitched monochromatic cry due to abnormal developmental of the larynx, broad nasal bridge, epicanthal folds, and severe psychomotor and mental retardation [2]. Most cases occur de novo [3,4], without the specific risk factors as an indication for invasive prenatal diagnosis. Therefore, it is difficult to make a prenatal diagnosis for a specific indication. According to previous reports, a structural abnormality may be present during an antenatal anatomical scan in some cases and may involve multiple systems, including the brain, heart, and genitourinary tract. Limited cases of prenatal diagnosis have been reported in the literature, because of non-specific ultrasound findings, such as CNS abnormality [5,6,7,8,9,10,11], cardiac defect [12], multiple anomaly [8,13,14], or fetal growth restriction [8]. In some cases, an abnormal level of beta-hCG, based on maternal serum screening or a positive cell-free DNA, provides the only clue to invasive prenatal diagnosis [3,8]. To the best of our knowledge, prenatal diagnosis of CdCS is still limited. Accumulation of cases with prenatal ultrasound findings in literature for further referencing and analysis is needed. Surprisingly, to date, no sonographic pattern specific to this disease has been systematically analyzed and proposed, even though several isolated cases have been dispersedly published in the literature. A sonographic pattern would warrant clinicians to conduct chromosome studies, especially in cases without indications, which occur predominantly, since 85–90% of cases occur de novo [8,15]. The objectives of this study are to present a unique case of CdCS, rarely prenatally as described before, and to perform an analytical review on prenatal sonographic features of the disease to identify the sonographic pattern specific to CdCS, which may be used for pattern recognition to facilitate early prenatal detection.

## 2. Case Presentation

**Figure 1:**
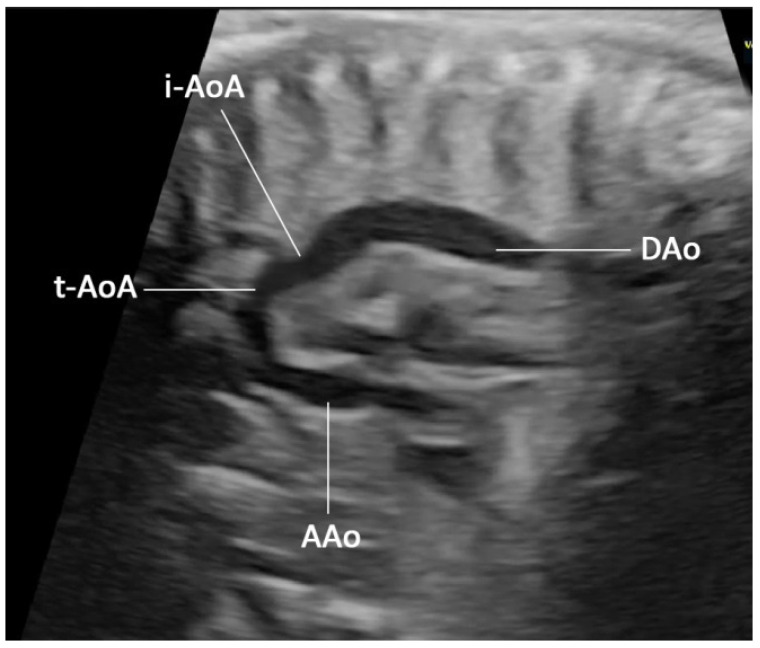
Prenatal ultrasound, sagittal scan, or long-axis view of the aorta shows a small aorta arch (tubular hypoplasia) connecting to the descending aorta with shelf appearance. (AAo: ascending aorta; i-AoA: isthmic aortic arch; t-AoA: transverse aortic arch; DAo: descending aorta).

**Figure 2:**
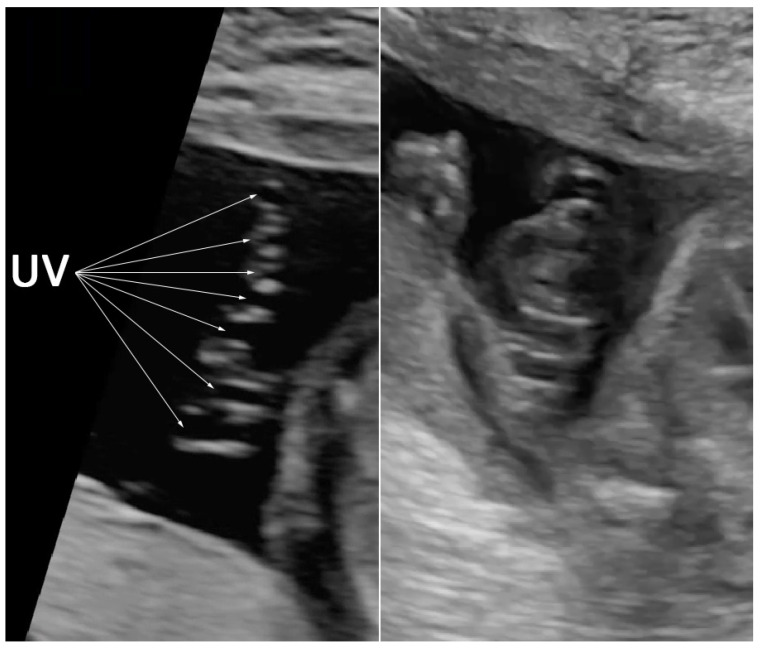
Prenatal ultrasound shows hypercoiling of the umbilical cord (7 loops in a 6 cm length) (UV: umbilical vein).

**Figure 3:**
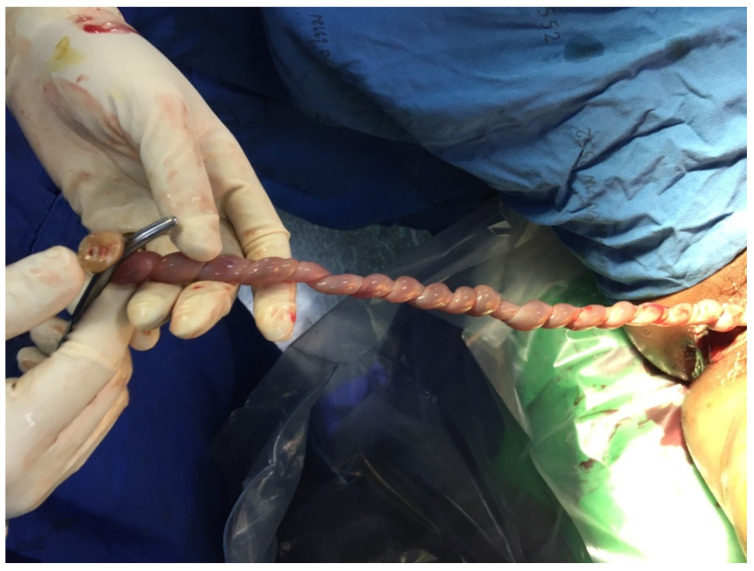
Postnatal finding shows hypercoiled umbilical cord.

**Figure 4:**
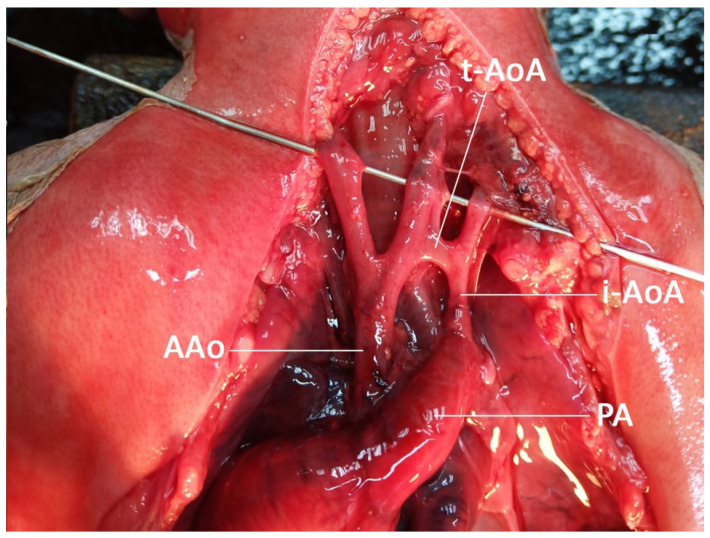
Autopsy finding shows coarctation of the aorta with tubular hypoplasia. (AAo: ascending aorta; i-AoA: isthmic aortic arch; t-AoA: transverse aortic arch; PA: pulmonary artery).

The case presentation was ethically approved by the institutional review board (Faculty of Medicine, Chiang Mai University), and written informed consent was obtained. A 36-year-old hill-tribe woman, G2P1001, attended her first antenatal-care visit at a public hospital at eight weeks of pregnancy. Her first pregnancy was uneventful, and the baby was healthy. She had chronic hypertension, diagnosed one year prior to the current pregnancy, and it was well-controlled with oral antihypertensive drugs (methyldopa). The patient and her husband were non-consanguineous. No teratogen exposure was recorded, and there was no known medical illness and genetic disorder in the family. She had a higher risk of development of preeclampsia because of advanced maternal age and chronic hypertension. Therefore, she took aspirin for preeclampsia prevention from 16 weeks onward. Her chronic hypertension was well-controlled with a single-agent hypertensive drug (methyldopa). Maternal serum screening for Down syndrome was not done but an amniocentesis for a chromosome study was performed at 16+ weeks of gestation due to an advanced maternal age. The ultrasound finding at that time was unremarkable. The karyotype was 46, XX, del (5p). At 20+ weeks of gestation, the fetal ultrasound (a real-time scanner GE Voluson E10; GE Healthcare Milwaukee, WI, USA, equipped with a transabdominal curvilinear transducer of frequency 3.5 MHz) showed cerebellar hypoplasia (trans-cerebellar diameter; TCD: 16.5 mm, <5th percentile for 20th week; 5th and 50th percentiles: 19.10 mm and 20.80 mm, respectively) with normal vermis and cisterna magna, ventriculomegaly (15 mm), peri-membranous VSD (4 mm), and coarctation of the aorta with tubular hypoplasia (Figure 1). The hypercoiled umbilical cord (1.1 coil/cm) was noted (Figure 2). After counseling, the couple chose to continue with the pregnancy. On the follow-up ultrasound at 34+ weeks, the fetal biometry, and growth were appropriate for her gestational age, but hypoplasia of the cerebellum and coarctation of the aorta with tubular hypoplasia still persisted. The pregnancy course was uneventful. No other medication apart from iron and vitamin supplementation, as standard antenatal care, was used during pregnancy. Preeclampsia and other obstetric complications were not observed. At 39 weeks of gestation, she had a decreased fetal movement. The ultrasound showed an undetectable heartbeat, generalized subcutaneous edema, pericardial effusion, and pleural effusion. Spontaneous labor occurred and resulted in a vaginal delivery, resulting in the birth of a stillborn female baby, weighing 2360 gm (just below the 5th percentile of the WHO growth chart) with a small placenta (300 gm). Grossly, the baby had desquamation, low-set ears, and micrognathia. It is worthy of note that the newborn had a hypercoiling umbilical cord (Figure 3). The autopsy findings included closed skull sutures, marked pleural and pericardial effusion, marked ascites, two lobes of the right lung, coarctation of the aorta with tubular hypoplasia (Figure 4), peri-membranous VSD, an abnormal position of the cecum, and an appendix position at the right upper quadrant of the abdomen.

## 3. Literature Review and Analysis

### 3.1. Methods

This is a narrative review focusing on prenatal ultrasound findings. The authors digitally searched and reviewed published articles concerning prenatal features of fetal CdCS. A search strategy was used to identify peer-reviewed manuscripts published between January 1990 and December 2021, using the following databases: PubMed, SCOPUS, and Web of Science. The article types included case reports, case series, original researches, and reviews. The search was updated in December 2021. Two authors independently assessed the title, abstract, and fulltext of the studies based on the keywords: (Cri-du-Chat [title] OR chromosome 5p- [title] OR del (5p)) AND (ultrasound OR sonographic OR ultrasonographic OR prenatal OR fetal). A total of 31 full-text articles were found and successfully extracted. After a thorough review, only 19 articles described prenatal sonographic features of CdCS and were included in the analysis. The data of baseline characteristics and ultrasound findings were extracted and pooled for analysis.

Statistical analysis: Descriptive analysis was performed using the statistical package for the social sciences (SPSS) software version 26.0 (IBM Corp. Released 2019. IBM SPSS Statistics for Windows, Version 26.0, IBM Corp, Armonk, NY, USA). Each sonographic finding was counted and presented as a frequency.

### 3.2. Result

Medical reports on prenatal diagnosis of fetal CdCS, including sonographic features, were systematically reviewed [3,5,6,7,8,9,10,11,12,13,14,15,16,17,18,19,20,21,22]. Nineteen studies were included, consisting of 47 cases with prenatal sonographic features. Various ultrasound findings were extracted from each article and systematically grouped. Calculations were done to obtain the prevalence of each feature, as presented in Table 1. Among the cases, 87.2% had at least one abnormal ultrasound finding, whereas no abnormal ultrasound finding was documented in about 13% of the cases. More than half of them (25 out of 47 cases) had multiple abnormal findings. Most cases had more than one feature. The cumulative data suggest that CdCS is associated with a specific pattern of sonographic features, which are unlikely to be just coincidental findings. The most consistent findings that are associated with the syndrome are cerebellar hypoplasia (29.8%), followed by cardiac abnormalities (19.1%), hydrops fetalis/fluid collection (17.0%), ventriculomegaly (14.9%), choroid plexus cyst (12.8%) and nasal bone hypoplasia (12.8%). Increased nuchal translucency/nuchal fold thickness is also common.

## 4. Discussion

Interestingly, CdCS has been long recognized and several studies have documented prenatal sonographic features of the disease. Nevertheless, the findings of individual cases are widely scattered in the literature. This is the first study aimed at compiling and organizing the various findings to create a specific pattern of prenatal ultrasound consistent with CdCS. Pattern recognition of the consistent findings, such as cerebellar hypoplasia (approximately one-third), cardiac anomaly, hydrops fetalis/fluid collection, ventriculomegaly, VSD, choroid plexus cyst (CPC), nasal bone hypoplasia, or increased nuchal fold/NT—especially their combination—should alert clinicians to counsel the couple regarding a chromosome study. This seems to be worthwhile, especially in cases with no obvious indications for invasive prenatal diagnosis. Interestingly, coarctation of the aorta and hypercoiling cord demonstrated in this case that it may be associated with CdCS and have never been described elsewhere. We believe that the pattern recognition would be helpful and facilitate early detection of CdCS, especially in cases of pre-viable stage. In the past, prenatal diagnosis of CdCS is usually unexpected and based on a chromosome study indicated by various risk factors, not a specific risk for CdCS. However, increased awareness of pattern recognition proposed by this review should be considered as a new indication for a chromosome study for CdCS.

According to this literature review, the most common fetal structural abnormalities detected by prenatal ultrasound include CNS anomaly, especially cerebellar hypoplasia, lateral ventriculomegaly, and CPC [6,7,8,9,10,13,15,18,19,22]; the anomalies may be isolated or combined or they may be combined with an abnormal serum marker. The congenital heart defect was found in about 15–20% [22,23] of CdCS. In prenatal studies, a common cardiac defect is VSD [8,13,22]. Other ultrasonographic findings are described in Table 1.

An abnormal serum marker is another indication of prenatal diagnosis, either a high risk of common aneuploidy [8,15,19,21] or isolation of an abnormal level of a biochemical. The profile of abnormal biochemistry in CdCS is varied, including high hCG [6,7,10,12], low hCG [3,18], high AFP [9], low AFP [21] or low PAPPA [15,19]. A discordant serum marker level can be explained by the parental origin, size, and specific area of haploinsufficiency, or other factors, such as epigenetic mechanism. Either cytokines, hormones, or growth factors can influence the gene encoding these biomarkers [3]. Likewise, a high risk of aneuploidy suggested by cfDNA testing can be the clue for diagnosis [8,19,22], with a wide range of positive predictive values for microdeletion detection [24]. However, the differences in test coverage, technical variability, size of microdeletion, and fetal fraction influence the efficacy of cfDNA. Until now, using the cfDNA as microdeletion screening is not yet recommended.

A wide variation of CdCS features may be explained by the parental origin of a deleted chromosome. Previous studies showed that speech delay, facial dysmorphism, or a cat-like cry are related to the parental origin of a deleted segment [25,26]. Furthermore, the specific region of haploinsufficiency results in specific clinical features, for example, 5p15.3 for a cat-like cry and speech delay, and 5p15.2 for facial dysmorphism, microcephaly, and severe intellectual disability [8]. Such genetic variations may also influence the variety of prenatal sonographic features, as indicated in our review and mentioned above.

In the view of obstetricians, invasive diagnostic procedures in cases of high risk of aneuploidy, indicated by various tests like serum markers, cfDNA, or aneuploidy in a previous child, can also be a definite diagnostic tool for CdCS regardless of ultrasound findings [3,8,14,17,19,22]. Nevertheless, most cases of CdCS have no such indications, leading to a missed diagnosis. It is worthy of note that most sonographic features in CdCS are minor (cerebellar hypoplasia, mild ventriculomegaly, CPC, and VSD) and could be simply overlooked, thereby missing the diagnosis. Therefore, the sonographic pattern proposed here can increase awareness of CdCS and lead to a chromosome study or facilitate early detection, especially in cases without obvious indications for chromosome study. Accordingly, prenatal ultrasound is even more important in providing clues leading to early diagnosis.

The most common cardiac defect associated with CdCS is VSD, while other cardiac defects are rarely reported. Based on the literature review, only a case among the studies, reported by Su et al., was associated with abnormal heart valve morphology. To the best of our knowledge, our case is the first reported case involving coarctation of the aorta (tubular hypoplasia). Since CdCS and coarctation of the aorta are rare, their combination in the same case should raise the possibility of genetic association, although it is possibly coincidental. Hills et al. [23] reported 21 cases of postnatal CdCS associated with a cardiac defect, in a retrospective review of 98,422 cases of congenital heart diseases. Among them, VSD and patent ductus arteriosus were the most common, with frequencies of 6/21 and 6/21, respectively. Other abnormalities were tetralogy of Fallot (5 cases), pulmonary valve atresia with ventricular septal defect (2 cases), pulmonary valve stenosis (1 case), and double outlet right ventricle (1 case). Accordingly, we conclude that CdCS may be associated with various cardiac defects, including coarctation of the aorta, however, VSD in most cases.

Although most cases occur de novo, such that the recurrence risk in a subsequent pregnancy is less than 1%, 10–15% of CdCS is caused by parental translocation of chromosome 5 with another chromosome [8,15]. Accordingly, after prenatal diagnosis of CdCS, a parental karyotype workup is essential for determining the recurrent risk. If there is a translocation in parents, the recurrent risk can be as high as 8.7–18.8% [2], which is important for genetic counseling and family planning.

## 5. Conclusions

In conclusion, the new knowledge gained from this study is that coarctation of the aorta and a hypercoiling umbilical cord are our unique observations and may possibly be associated with CdCS, though the mechanism is unclear. More importantly, we performed an analytical review and propose a sonographic pattern relatively specific to CdCS, which may be useful in clinical practice to warrant chromosome study. Pattern recognition of findings like cerebellar hypoplasia, VSD, ventriculomegaly, CPC, nasal bone hypoplasia, or increased nuchal fold/NT, especially in their combination, should alert clinicians to counsel the couple for chromosome study.

## Figures and Tables

**Table 1:** Sonographic features of 47 fetuses with Cri-du-Chat syndrome [3,5,6,7,8,9,10,11,12,13,14,15,16,17,18,19,20,21,22].

No.	Findings	N (47)	%
1	Cerebellar hypoplasia	14	29.8
2	Cardiac anomaly (VSD, others)	9	19.1
3	Hydrops fetalis (fluid collection)	8	17.0
4	Ventriculomegaly	7	14.9
5	Choroid plexus cyst (CPC)	6	12.8
6	Nasal bone hypoplasia	6	12.8
7	Abnormal growth	4	8.5
8	Increased nuchal fold thickness (INF)	4	8.5
9	Increased nuchal translucency thickness (INT)	4	8.5
10	Dilated cisterna magna	3	6.4
11	Clubfoot	2	4.3
12	Hypospadias	2	4.3
13	Neural tube defect (NTD)	2	4.3
14	Pyelectasis	2	4.3
15	Single umbilical artery (SUA)	2	4.3
16	Agenesis of corpus callosum	1	2.1
17	Cystic adenomatoid malformation	1	2.1
18	Cystic hygroma	1	2.1
19	Dandy-Walker syndrome	1	2.1
20	Facial cleft	1	2.1
21	Interrupted inferior vena cava	1	2.1
22	Microretrognathia	1	2.1
23	Negative	6	12.8

## Data Availability

The data of this report are available from the corresponding authors upon request.
